# Structural and functional comparative analysis of porcine and human salivary glands: a narrative review for translational research

**DOI:** 10.1007/s10735-025-10678-y

**Published:** 2026-01-17

**Authors:** Lara Vanelli, Giovanna P. Florezi, Cristina Massoco, Silvia Vanessa Lourenço

**Affiliations:** 1 Stomatology Department, Faculdade de Odontologia da Universidade de São Paulo, São Paulo, São Paulo Brasil; 2 Laboratory of Comparative Imuno-Oncology, Department of Pathology, Faculdade de Medicina Veterinária da Universidade de São Paulo, São Paulo, São Paulo Brasil; 3 Surgery Department, Faculdade de Medicina da Universidade de São Paulo, São Paulo, São Paulo Brasil

**Keywords:** Salivary glands, Animal models, Pigs, Saliva, Xerostomia, Comparative anatomy

## Abstract

The use of animal models plays a crucial role in advancing our understanding of human diseases, providing valuable insights into pathophysiological mechanisms and supporting the development of innovative therapeutic strategies. Among the available models, porcine models have been recognized as particularly relevant due to their anatomical, genetic, and physiological similarities to humans. Notably, this review provides the first comprehensive comparative analysis of porcine and human salivary glands, integrating anatomical, functional, and translational perspectives. Porcine salivary glands offer a promising platform for comparative studies, as they share both structural and functional characteristics with human salivary tissue. This review highlights the anatomical and functional parallels between porcine and human salivary glands, emphasizing their significance in biomedical research. In recent years, porcine models have been increasingly employed in studies investigating disease mechanisms, drug efficacy and toxicity, as well as regenerative medicine. The integration of these models into translational research is expected to facilitate the development of novel therapeutic approaches and improve clinical outcomes in human patients. Ongoing research on the application of porcine models remains essential for refining current methodologies and expanding the scope of salivary gland studies related to human health and disease.

## Introduction

Salivary glands play a crucial role in oral health, being responsible for the production and secretion of saliva into the oral cavity. Saliva is a multifunctional fluid with lubricating properties that protect the oral mucosa and dental structures, aid in speech, digestion, and swallowing, among other functions (Shang et al. 2023). In humans, there are three bilateral pairs of major salivary glands (MSGs): the parotid (PG), submandibular (SMG), and sublingual glands (SLG). In addition to the major salivary glands, numerous minor salivary glands are distributed throughout the oral cavity (Tucker 2010; Ogle 2020).

The literature consistently reports that various factors can disrupt salivary gland homeostasis and, consequently, impair their function. Examples include tumors, lifestyle factors such as smoking and alcohol consumption, viral infections, diabetes, autoimmune diseases, menopause, and aging (Miranda-Rius et al. 2015; Bologna et al. 2020; Zięba et al. 2022; Piacenza Florezi et al. 2024). One of the most common resulting symptoms is xerostomia, defined as the subjective sensation of dry mouth. Although not life-threatening, xerostomia significantly compromises quality of life and affects both oral and systemic homeostasis (Bologna et al. 2020; Proctor and Shaalan 2021). Importantly, xerostomia can occur even in the absence of an objective reduction in salivary flow (hyposalivation), indicating that its underlying causes extend beyond quantitative changes in saliva production and may involve alterations in its lubricating properties (Sardellitti et al. 2024). A relationship has been suggested between saliva secreted by the minor salivary glands and the sensation of dry mouth, which may be associated with alterations in mucin concentration, a glycoprotein that plays a fundamental role as a lubricant in the oral cavity (Satoh-Kuriwada et al. 2012). This underscores the relevance of qualitative changes in salivary composition or function, highlighting that salivary gland impairments can arise from multiple mechanisms (Proctor and Shaalan 2021). For the past 15 years, our group has been actively engaged in salivary gland research, advancing the understanding of degenerative conditions affecting these glands and their clinical implications, while conducting basic and applied studies to elucidate salivary gland physiology from their morphogenesis to the pathophysiological repercussions of conditions and diseases that lead to xerostomia, highlighting in particular the role of aquaporins in mediating the interaction between salivary glands and the vasculature for saliva production.

In biomedical research, rats and mice are widely used as animal models to investigate metabolic disorders, cancer, behavior and addiction, as well as salivary gland dysfunction (May et al. 2015; Fujiwara 2018; Smith et al. 2019). In addition to sharing some anatomical and functional similarities with humans, these animal models are easily affordable and simple to manage (Li et al. 2007). Both possess three pairs of major salivary glands (PG, SMG, and SLG) and have ductal systems composed of intercalated (ID), striated (SD), excretory (ED), and main excretory ducts. However, histological differences exist (Amano et al. 2012). For instance, the human parotid gland contains well-defined intralobular adipose tissue, whereas adipocytes are not prominent in the parotid gland of rodents. Additionally, the human submandibular gland is a mixed gland containing both serous and mucous acini, while in rodents it is exclusively serous (Amano et al. 2012). Beyond histology, differences in organ size and weight, coupled with the short lifespan of rodents, limit their suitability for studying long-term effects and developing preclinical therapies.

**Fig. 1:**
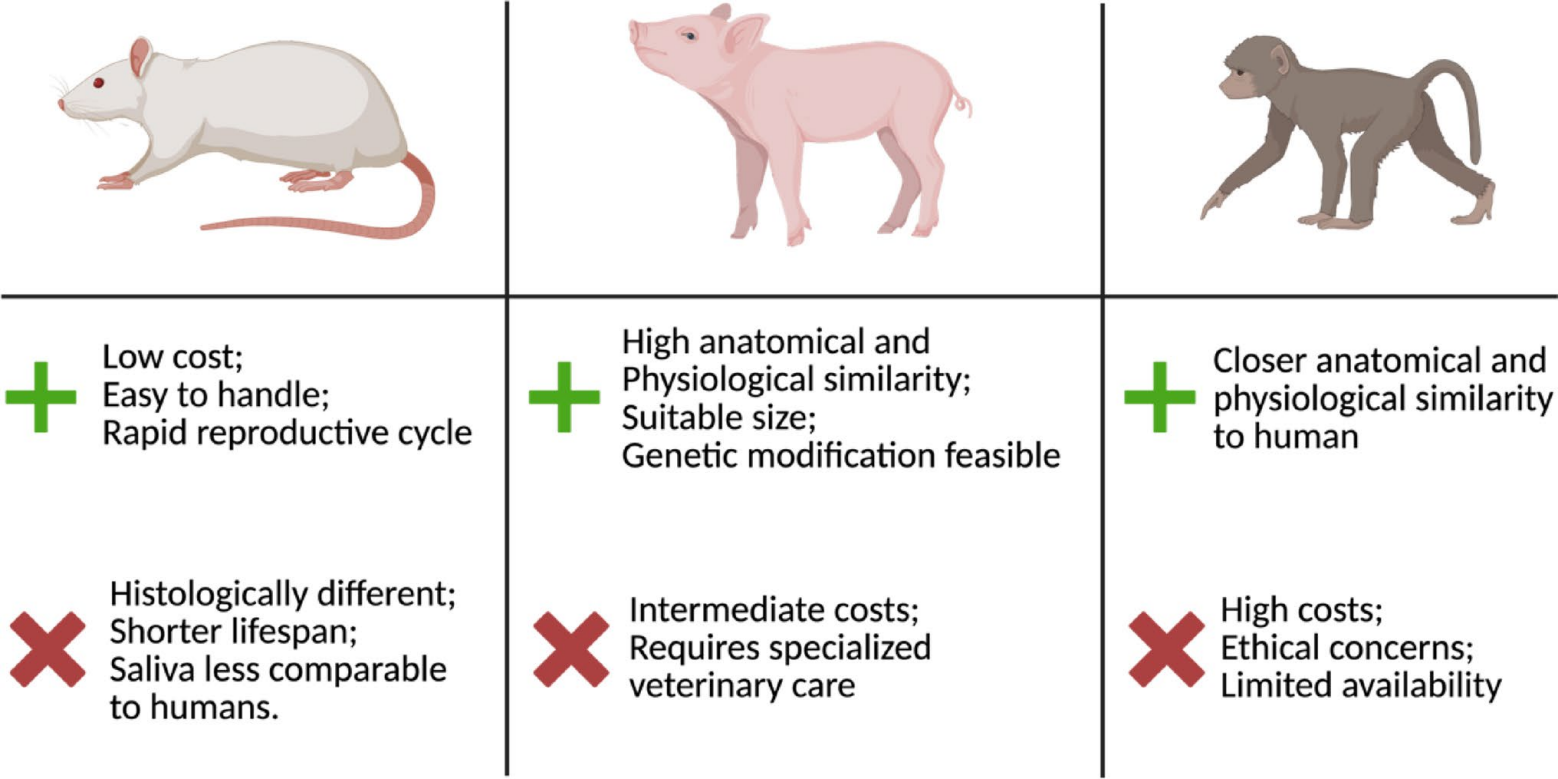
Comparison of animal models for salivary gland research

Non-human primates, on the other hand, offer the closest anatomical and physiological similarity to humans, making them highly predictive for clinical outcomes. However, their use is constrained by high costs, ethical considerations, and limited availability (Carvalho et al. 2018). Porcine represent an intermediate model, combining practical advantages with substantial anatomical and physiological resemblance to humans, thereby offering a valuable platform for translational salivary gland research, as shown in Fig. 1 (Radfar and Sirois 2003; Meurens et al. 2012; Joshi et al. 2024).

Over the past two decades, pigs have emerged as valuable models for biomedical research due to their high genetic homology with humans, anatomical and physiological similarities, and susceptibility to certain human diseases (Luo et al. 2012). They also share important oral features with humans, such as mucosal structure and dentition, including both deciduous and permanent teeth with comparable development and eruption patterns (Li et al. 2004; Wang et al. 2007) Their large body size enables safer and more accurate drug dosing, while similarities in organ size and their longer lifespan compared to rodents enhance translational relevance. Moreover, pigs can be genetically modified to replicate the genetic and/or functional basis of specific human diseases, enabling the development of refined and clinically relevant models (Flisikowska et al. 2014; Perleberg et al. 2018; Kalla et al. 2020). For example, Stoltz et al. (2010) demonstrated that pigs genetically engineered to develop cystic fibrosis recapitulate key respiratory manifestations of the disease observed in humans (Stoltz et al. 2010). Similarly, transgenic pigs carrying mutations associated with Alzheimer’s disease have been established to reproduce amyloid pathology and cognitive decline (Jakobsen et al. 2016). These examples highlight the versatility and translational relevance of genetically engineered pigs.

Despite the recognized advantages of pigs as biomedical models, studies using them to investigate salivary gland diseases remain limited. This underrepresentation is partly due to the historical focus on systemic and neurological conditions, leaving pigs salivary research largely unexplored. Therefore, the aim of this article is to provide a critical, narrative review of the literature comparing porcine and human salivary glands and saliva composition under both healthy and pathological conditions. By highlighting the structural and functional similarities between pig and human salivary glands, this review underscores the pig’s relevance as an experimental model and emphasizes the need for further research to fully exploit its translational potential in understanding and treating salivary gland disorders. Addressing this gap could advance the development of clinically relevant interventions for salivary gland dysfunctions.

## Methodology

This review followed a narrative approach with structured literature screening. Relevant studies were identified through searches in scientific databases, primarily PubMed, using combinations of keywords such as salivary glands, pigs’ model, comparative anatomy, and xerostomia. Only articles published in English were considered.

**Fig. 2:**
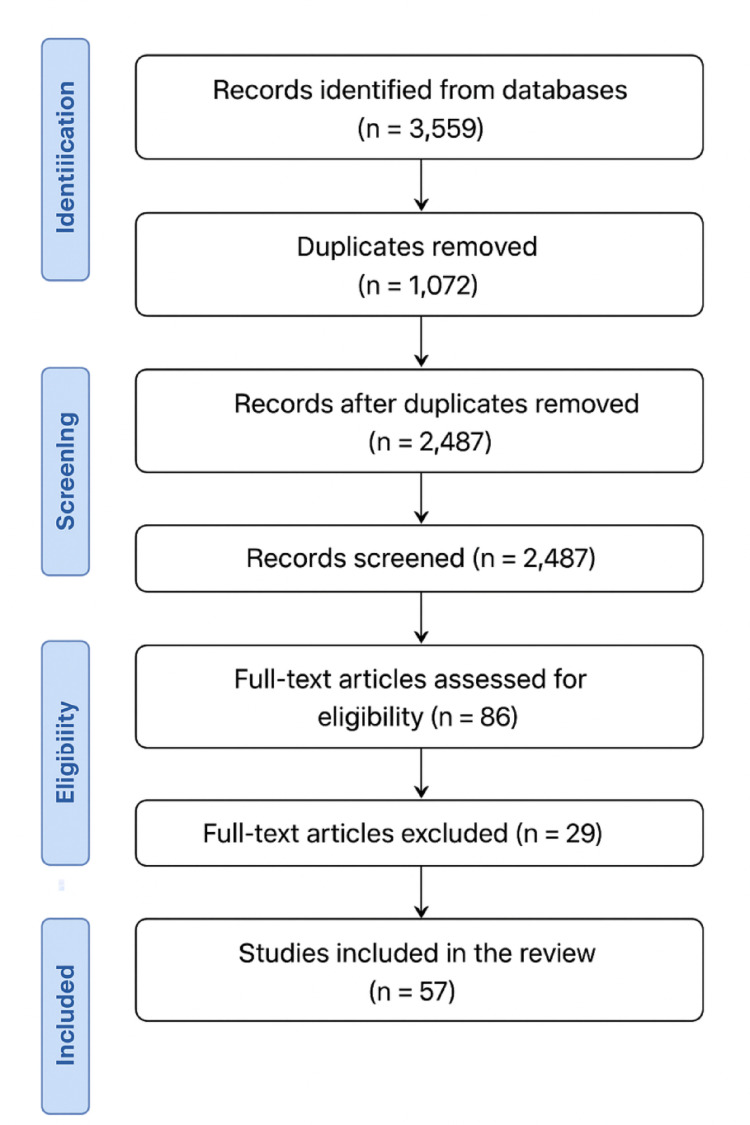
PRISMA-like flow diagram summarizing the selection process of the studies included in the review

A total of 3,559 records were initially retrieved and screened using the Rayyan platform. After removal of 1,072 duplicates, 2,487 records were screened by title and abstract. Following eligibility assessment of 86 full-text articles, 57 studies were included in the final synthesis (Fig. 2). Studies were selected based on their relevance to the structural, functional, molecular, or translational aspects of porcine and human salivary glands. No systematic review protocol or meta-analysis was applied. The selected references were critically analyzed and thematically organized to emphasize anatomical similarities, physiological functions, and the biomedical applications of porcine salivary glands in human disease research.

A total of 3,559 records were imported from databases. After removing 1,072 duplicates, 2,487 articles were screened by title and abstract. Of these, 2,401 were excluded, and 86 full-text articles were assessed for eligibility. Finally, 57 studies met the inclusion criteria and were incorporated into the narrative synthesis.

### Porcine and human craniofacial anatomy and development

Porcine share several facial anatomical structures with humans, including the nasal bone, zygomatic bone, orbit, frontal bone, mandible, and maxilla (Štembírek et al. 2012). The pigs oral cavity differs from that of humans in being narrower and more elongated, whereas humans present a more oval shape. As in humans, the pigs buccal and labial mucosae are smooth and lack papillae, and the cheeks are covered by a thick layer of fat. The pigs tongue is firmly attached to the floor of the oral cavity by a double frenulum, with a bifurcated lingual frenulum, differing from human anatomy. Despite these anatomical distinctions, the histological structure of the palatal and lingual mucosa in pigs exhibits patterns similar to those found in humans, supporting their relevance as a comparative model (Štembírek et al. 2012).

In 2010, Zhou et al. investigated the development of salivary glands in minipigs using histological and ultrastructural analyses (Zhou et al. 2010). They identified five distinct developmental stages, corresponding to those described for the mouse submandibular gland and comparable to stages observed in humans (Tucker 2007; Teshima et al. 2016; Lourenço et al. 2007). At 40 days of embryonic development, epithelial cell proliferation initiated the formation of the initial buds of the submandibular and parotid glands. By 60 days, a more defined cellular organization was evident, with both glands displaying a similar structure, although the parotid gland had fewer primordial acini. At 80 days, the ductal system of the submandibular gland was formed, and loose connective tissue with abundant blood vessels was observed. The overall shape and structure of both glands remained similar, but the parotid gland still presented fewer acini and relatively poor cellular differentiation. At 95 days, all ductal levels of the submandibular gland were differentiated and undergoing maturation. The ducts and acini of the parotid gland were histologically similar to those of the submandibular gland, but its acini were exclusively serous. On postnatal day 0, the glandular structures were nearly mature and resembled those of adult animals. All ductal levels were differentiated and continued to mature, with the glandular lobes of the submandibular gland well defined, whereas the striated and intercalated ducts of the parotid gland were not yet clearly distinguishabled (Zhou et al. 2010; Wells and Patel 2010).

### Anatomical, morphological, and functional aspects of the swine salivary glands

Porcine and humans present a set of three MSGs. In pigs, the parotid gland is relatively large, triangular in shape, and covered by adipose tissue. Its excretory duct passes through the buccinator muscle, in the region between the premolar and first molar teeth, and opens into the oral vestibule via the parotid papilla (Boshell and Wilborn 1978; Štembírek et al. 2012). Wang et al. (1998) demonstrated that the parotid gland of minipigs shares several anatomical and physiological features with its human counterpart, notably the volume and diameter of the main excretory duct. These similarities are particularly relevant for performing sialography in minipigs (Wang et al. 1998).

The submandibular glands (SMGs) are enclosed by a compact capsule and exhibit an ovoid shape. They are located superficially to the suprahyoid and infrahyoid muscles and are overlapped by the parotid gland (PG). Their ductal system originates at the anterior border of the gland and extends forward and upward through the intermandibular space, running beneath the mylohyoid muscle. The main duct passes through the sublingual gland (SLG) duct and terminates at the sublingual papilla (Zhang et al. 2005; Štembírek et al. 2012).

Pigs possess two sublingual glands. The monostomatic SLG consists of multiple ducts that converge into a single major sublingual duct, which opens into the oral cavity at the same site as the mandibular duct. In contrast, the polystomatic SLG—larger in size—secretes directly into the sublingual recess through multiple small openings (Štembírek et al. 2012).

In the terminal portion of the PG, the acinar cells are predominantly serous, whereas in the SMG and SLG, the parenchyma consists of mixed serous and mucous acini (Štembírek et al. 2012; Zhang et al. 2018). This pattern is also observed in the corresponding human MSGs (de Paula et al. 2017).The ductal system of the SMG in both pigs and humans displays similarities, including the presence of intercalated, striated, and excretory ducts, none of which contains secretory granules (Zhang et al. 2018).

As in humans, minor salivary glands are distributed throughout the pigs oral cavity, including the buccal mucosa and the labial, palatal, and lingual regions (Štembírek et al. 2012; Prims et al. 2019; Aframian et al. 2019; Li et al. 2025).

Molecular features of pigs salivary glands have not yet been thoroughly explored. However, Zhang et al. (2018) demonstrated the presence of claudin-1 to claudin-12, occludin, and zonula occludens-1 (ZO-1) proteins in the major salivary glands of pigs and humans using reverse transcription polymerase chain reaction (RT-PCR). Additionally, immunofluorescence analysis revealed that claudins 1 and 3, occludin, and ZO-1 are present in the acinar and ductal cells of the SMG in both species. In the same study, three-dimensional (3D) imaging of tight junction (TJ) proteins in the SMG showed that these structures form a honeycomb-like pattern on the luminal surface of the ducts, whereas in the acini they display an irregular distribution (Zhang et al. 2018).

The claudin family represents the main structural component of TJs and plays a fundamental role in the paracellular barrier function of these junctions. To date, 27 claudins have been identified in humans (Tanaka et al. 2017; Tsukita et al. 2019; Citi et al. 2024). Lourenço et al. (2007), using histochemical and immunofluorescence techniques, reported the expression of claudins 1, 3, 4, 5, 7, and 11 at different stages of human salivary gland development, from early formation to full maturation (Lourenço et al. 2007). Furthermore, certain claudins may participate in the formation of paracellular channels, enabling the selective passage of water, ions, and small solutes through the extracellular space (Coutinho-Camillo et al. 2011; Günzel and Yu 2013; Tanaka et al. 2017; Tsukita et al. 2019; Citi et al. 2024; Samiei et al. 2019).

Recent evidence further highlights the functional relevance of claudins in salivary gland physiology. Experimental studies have shown that mutations in claudin genes or disruptions in tight junction organization can directly impair salivary secretion. In particular, claudin-10b mutations are responsible for HELIX syndrome, which includes xerostomia, hypohidrosis, electrolyte imbalance, lacrimal gland dysfunction, and ichthyosis (Jo et al. 2022). Similarly, alterations in claudin expression patterns have been associated with secretory dysfunction in IgG4-related sialadenitis, including downregulation of claudin-3, -4, -6, -7, -8, -10, and -12, as well as occludin and ZO-1. These changes, together with mislocalization of tight junction proteins and disorganization of the actin cytoskeleton, have been linked to reduced salivary flow (Min et al. 2020). These findings underscore the critical role of claudins in saliva secretion and suggest that they may serve as potential targets for novel therapeutic strategies. Additionally, the molecular architecture of porcine salivary glands closely resembles that of human glands, supporting the pig as a relevant translational model; however, the complete repertoire of surface proteins and phenotypic traits remains to be fully elucidated (Table 1).

**Table 1:** Comparative morphological and functional features of human and porcine salivary glands

Feature	Human salivary glands	Porcine salivary glands	References
Major glands	Parotid, submandibular, sublingual	Same three major glands	Štembírek et al. (2012), de Paula et al. (2017)
Minor glands	Scattered in mucosa; active from birth	Same locations; buccal glands in two dorsal/ventral rows	Štembírek et al. (2012), de Paula et al. (2017)
Developmental timing	4th–12th embryonic week; minor glands start earlier	Parotid develops ~ 15 days after SMG; stages: placode → bud → branching → canalization → maturation	Zhou et al. (2010), Porcheri and Mitsiadis (2019)
Ultrastructural findings (parotid)	Pyramidal acinar cells with basally located nuclei, abundant rough ER, Golgi and secretory granules	Pyramidal serous acinar cells, spherical basal nuclei, cytoplasm packed with secretory granules; myoepithelial cells between basal part and basal lamina	Wang et al. (1998)
Parotid gland acini	Serous	Serous	Zhou et al. (2010), de Paula et al. (2017)
Submandibular gland acini	Mixed (serous > mucous)	Mixed (serous + mucous)	Zhou et al. (2010), de Paula et al. (2017)
Sublingual gland acini	Mucous-predominant mixed	Mucous-predominant mixed	Zhou et al. (2010), de Paula et al. (2017)
Parotid duct length (mm)	50–70	111–152	Wang et al. (2007)
Submandibular duct length (mm)	~ 50	105–140	Wang et al. (2007)
Ductal system	Intercalated > striated > excretory	Same structure	Zhou et al. (2010), de Paula et al. (2017)
Branching duct pattern (parotid)	Multiple branches from main duct, variable pattern	4–6 branching ducts from inferioposterior margin of distal 1/3 of main duct	Wang et al. (1998)
Tight junction protein expression	Claudin-1, 3, 4, occludin, ZO-1 in acinar and ductal cells	Claudin-1, 3, 4, occludin, ZO-1 in acinar and ductal cells; similar distribution to human	Zhang et al. (2018)

### Salivary flow, composition, and proteomic approaches

The pairs of major salivary glands (MSGs) are responsible for producing and secreting approximately 90% of the total saliva volume. The minor salivary glands—ranging from 600 to 1,000 in number and distributed throughout the oral cavity—account for the remaining 10%. This secretion plays a crucial role in oral lubrication due to its protective and mucous componentes (Patel et al. 2006; Holmberg and Hoffman 2014; de Paula et al. 2017). Similarly, the relative contribution of each gland type to saliva production in pigs follows the same pattern (Prims et al. 2019). Furthermore, studies comparing parotid and mixed saliva flow rates in pigs and humans found no significant differences, reinforcing the suitability of porcine models for salivary gland research (Li et al. 2007; Wang et al. 2007; Štembírek et al. 2012).

Saliva is a complex biological fluid composed predominantly of water (approximately 99%), along with proteins and various organic and inorganic compounds. Among the inorganic components, sodium, potassium, calcium, chloride, magnesium, and carbonates are most abundant. The organic fraction includes enzymes such as amylase, peroxidase, and lipase, as well as mucins, lysozyme, lactoferrin, kallikrein, cystatins, hormones, and growth factors. In healthy individuals, the daily salivary secretion ranges from 0.5 to 1.5 L (Chicharro et al. 1998; Kaczor-Urbanowicz et al. 2017; Martina et al. 2020).

Li et al. (2007) conducted a comparative analysis of salivary flow, pH, buffering capacity, and biochemical composition in minipigs, rats, and humans. The study demonstrated that humans and minipigs exhibit similar total salivary flow, whereas rats displayed distinct values, reflecting their lower physiological similarity to humans. Regarding salivary pH and buffering capacity, minipigs presented higher values than humans, while the differences between rats and humans were even more pronounced. Biochemical analysis revealed that the profiles of Na⁺, K⁺, and Ca2⁺ in minipigs more closely resembled those of humans than those of rats. However, in the case of amylase activity, minipigs showed substantially higher levels compared to humans, indicating a relevant metabolic peculiarity (Li et al. 2007).

Beyond the physicochemical characterization of saliva, proteomic profiling further emphasizes the translational relevance of the porcine model. The majority of salivary proteins originate from the salivary glands, whereas a minor fraction is derived from plasma, comprising potential biomarkers of diagnostic value. Owing to its non-invasive nature, low cost, and ease of collection and processing, saliva represents a promising diagnostic fluid (Gutiérrez et al. 2011, 2013; Lamy and Mau 2012; Zhang et al. 2013; Cuevas-Córdoba and Santiago-García 2014). Mammalian saliva is predominantly composed of α-amylase, proline-rich proteins, histatins, cystatins, and statherin, in addition to mucins, immunoglobulins, carbonic anhydrase, and lactoperoxidase (De Sousa-Pereira et al. 2013).

Gutiérrez et al. (2011) reported mean protein concentrations of 0.68 mg/mL in control animals and 2.4 mg/mL in subclinically infected pigs (Gutiérrez et al. 2011). Notably, several proteins commonly present in human saliva were also detected in pigs, including amylase, immunoglobulins (IgA, IgG), carbonic anhydrase VI, lipocalins, and cystatins, underscoring the model's translational relevance (Gutiérrez et al. 2011; Gutierrez et al. 2014; Prims et al. 2019). Moreover, acute-phase proteins such as haptoglobin and C-reactive protein exhibited significantly elevated levels in infected animals, supporting their potential utility as salivary biomarkers of health and disease (Gutiérrez et al. 2011; Gutierrez et al. 2014).

Prims et al. (2019) characterized the gland-specific salivary proteome of pigs and reported that parotid saliva exhibited a higher protein concentration compared to saliva secreted by the mandibular and sublingual glands. Although flow rates did not differ significantly between glandular secretions, parotid saliva was notably richer in proteins. This pattern is consistent with that observed in humans, where parotid saliva displays higher protein content regardless of stimulation. In contrast, studies in humans and rodents have shown that the combined flow of mandibular and sublingual glands tends to exceed that of the parotid gland. The absence of this difference in pigs, as reported by Prims et al. (2019), may be attributed to the use of anesthetics during sampling, the nature of the applied stimuli, or intrinsic physiological characteristics of the species. Further investigations are required to clarify these potential interspecies differences in salivary secretion (Prims et al. 2019).

Beyond the gland-specific characterization, proteomic investigations have further delineated the complexity and translational relevance of the porcine salivary proteome. Using a shotgun proteomic approach with isobaric labeling and high-resolution tandem mass spectrometry, Prims et al. (2019) identified 128 proteins in parotid and mandibular–sublingual secretions. Although both glands shared a largely overlapping protein repertoire, quantitative differences were observed: α-amylase and carbonic anhydrase VI were more abundant in parotid saliva, whereas lipocalin and submaxillary apomucin predominated in mandibular and sublingual secretions. These glandular expression patterns show trends similar to those described in humans, reinforcing the physiological resemblance between porcine and human salivary systems (Prims et al. 2019). Building on this foundation, Prims et al. emphasized the diagnostic and welfare-monitoring potential of the porcine salivary proteome, proposing proteins such as odorant-binding protein, chitinase, lipocalin-1, and α2-HS-glycoprotein as candidate biomarkers of stress and health status (Gutiérrez et al. 2013; Prims et al. 2024). Collectively, these findings underscore both the conserved molecular composition of mammalian saliva and the suitability of the pig as a translational model for proteomic and biomarker studies. These findings are summarized in Table 2.

**Table 2:** Comparative biochemical features of human and porcine salivary glands

Feature	Human salivary glands	Porcine salivary glands	References
Saliva pH	7.32 ± 0.17	7.77 ± 0.18	Li et al. (2007)
Whole saliva flow (mL/min)	1.13 ± 0.87	1.40 ± 0.39	Li et al. (2007)
Parotid saliva flow (mL/min)	0.35 ± 0.21	0.31 ± 0.38	Li et al. (2007)
Amylase activity (IU/L)	1.64 ± 1.00	1125.73 ± 22.73	Li et al. (2007)
Ion content	High Na + , low K + , Ca2 +	Low Na + , high K + , Ca2 +	Li et al. (2007)
Proteomic profile highlights	> 3000 proteins; α-amylase, mucins, proline-rich proteins, CA VI	128 identified proteins; CA VI, α-amylase higher in parotid; lipocalin, submaxillary apomucin higher in mandibular/sublingual	Prims et al. (2019)

Mucins are the predominant macromolecular components of normal, unstimulated saliva and can be divided into two main populations: MUC5B, a high-molecular-weight, polymeric, gel-forming mucin, and MUC7, a lower-molecular-weight, non-polymeric mucin. Their large size and the high density of negatively charged O-glycans confer crucial functions in the hydration and lubrication of oral surfaces. Moreover, mucins mediate interactions with bacteria via their glycans and protein domains, facilitating bacterial sequestration and contributing to oral defense mechanisms (Rousseau et al. 2008; Piras et al. 2011).

 Five human gel-forming mucins are described: MUC6, MUC2, MUC5AC, MUC5B, and MUC19. MUC5B is predominantly expressed in the mucous cells of the human salivary glands. Although the expression of MUC19, the most recently identified mucin, has not been fully characterized, it has been detected in human airways and salivary glands. In mice, the Muc19 gene has been fully sequenced and is strongly expressed in the sublingual and submandibular salivary glands. Furthermore, mRNA sequences of submaxillary mucins from pigs and cattle show high similarity to the mouse Muc19 sequence, suggesting that MUC19/Muc19 is likely present in the saliva of humans and other mammals (Rousseau et al. 2008).

These molecular and functional similarities strongly support the use of pigs as a translational model for human salivary mucin research. In particular, the predominance of MUC5B in both human and porcine salivary glands, together with the likely presence of MUC19, indicates that pigs provide a relevant system to study mucin biology, secretion mechanisms, and their critical roles in oral hydration, lubrication, and microbial defense.

### Porcine models in regenerative therapies and genetic engineering

Transgenic animal production has been feasible for over two decades. Historically, microinjection was the most commonly used method, involving the injection of DNA, RNA, or proteins into embryos (Gün and Kues 2014; Yao et al. 2016). However, this approach is inefficient (2%–3% in pigs), time-consuming, and costly, as many embryos are required to generate a single transgenic animal. More recently, advanced techniques such as somatic cell nuclear transfer (SCNT), CRISPR/Cas9 genome editing, and viral vector-mediated gene transfer have been developed and refined (Jakobsen et al. 2013; Hryhorowicz et al. 2020; Maga et al. 2003; Wei et al. 2022).

Small rodent models are widely used in basic research, yet they present limitations in translating findings to humans. Pigs offer closer anatomical and physiological resemblance, making them ideal for translational studies, particularly when genetically modified to better mimic human conditions. Porcine models can be engineered to study human diseases, produce recombinant proteins at pharmaceutical scales, and provide tissues and organs for xenotransplantation (Matsunari and Nagashima 2009; Aigner et al. 2010; Flisikowska et al. 2014; Hryhorowicz et al. 2017; Perleberg et al. 2018; Petters 1994). For example, transgenic pigs have been used to express human proteins such as recombinant protein C, retaining equivalent biological activity to plasma-derived forms (Velander et al. 1992).

In salivary gland research, porcine models have been instrumental in studying radiation-induced hypofunction, a common side effect in head and neck cancer patients. Porcine salivary glands exhibit injury responses similar to humans, allowing investigation of tissue protection, regeneration, and functional restoration. (Radfar and Sirois 2003; Baum et al. 2006; Wang et al. 2015). Gene therapy strategies using adenoviral and adeno-associated viral vectors have successfully enhanced fluid secretion, preserved acinar and ductal cells, and improved local vascularization in irradiated glands (Baum et al. 2006; Gao et al. 2011; Wang et al. 2015). Additionally, CRISPR/Cas9-mediated genome editing has enabled the creation of tissue-specific transgenic models, such as pigs expressing digestive enzymes in salivary glands, supporting both translational and biotechnological applications (Li et al. 2020).

Salivary glands are among the most radiosensitive tissues in the body and exhibit low regenerative potential due to their composition of highly differentiated cells. Exposure to radiation during radiotherapy results in extensive damage to acinar cells, blood vessels, and surrounding nerves, leading to hyposalivation (Li et al. 2005). In patients with head and neck cancer undergoing radiotherapy, it is estimated that 40% to 60% develop irreversible xerostomia. Several approaches have been explored to promote the functional regeneration of salivary glands following radiation-induced damage. Recent studies have highlighted the potential of stem cells and three-dimensional biofabrication technologies in reconstructing the secretory acinar compartment. In this context, Phan et al. (2023) reviewed significant advances in 3D bioprinting platforms and culture systems that enable the formation of salivary gland organoids, emphasizing their potential applications in clinical transplantation, disease modeling, and high-throughput drug screening. The study also discusses the use of extracellular matrices derived from porcine tissues in magnetic bioprinting platforms, which provide a favorable microenvironment for cell differentiation and functionality, demonstrating the translational applicability of pigs-derived components in regenerative therapies (Phan et al. 2023).

Additionally, Wu et al. (2021) developed an immunosuppressed miniswine model to evaluate the feasibility and integration of human cell-based implants for the treatment of radiation-induced xerostomia. The authors implanted three-dimensional constructs composed of hyaluronate hydrogels containing human salivary stem/progenitor cells into the renal capsule and parotid gland. Their findings demonstrated high cell viability, biomaterial biocompatibility, and evidence of vascular and neural integration, with no signs of rejection under immunosuppression. Therefore, the immunosuppressed miniswine was proposed as a promising translational model for testing salivary cell-based therapies prior to clinical application in humans (Wu et al. 2021).

Hai et al. (2009) demonstrated that miniature pigs, through the use of adenoviral vectors, can serve as a valuable animal model for preclinical gene therapy studies aimed at correcting radiation-induced damage in the parotid gland. However, the authors also observed that, although AAV2 vectors enabled prolonged transgene expression in this model, the stability of expression was lower than that observed in rodents and primates, and the protein was unusually directed predominantly into saliva. These findings indicate that, despite the potential of the porcine model for certain studies, its physiological particularities may limit its application in the investigation of gene therapies for salivary glands (Hai et al. 2009). Conversely Liang Hu et al. (2021) demonstrated that the transient activation of Hedgehog signaling alleviated salivary hypofunction after radiation in porcine models by inhibiting radiation-induced cellular senescence and preserving both the parotid microvasculature and resident macrophages (Hu et al. 2021).

Hu et al. (2018) demonstrated that intraglandular delivery of the Sonic Hedgehog (Shh) gene alleviated radiation-induced hyposalivation in miniature pigs. The treatment improved salivary flow and preserved acinar cells, microvasculature, and parasympathetic innervation while reducing fibrosis and cellular senescence. These results provided the first large-animal evidence that Hedgehog pathway activation can restore salivary function after irradiation, reinforcing the translational potential of gene therapy approaches for xerostomia (Hu et al. 2018). Similarly, transplantation of autologous adipose-derived stem cells (ADSCs) has shown protective effects against radiation-induced salivary hypofunction in miniature pigs. ADSC treatment preserved acinar structures, improved salivary flow rate and amylase production, and increased microvessel density compared with irradiated controls, confirming the regenerative and angiogenic potential of stem cell-based therapy in a large-animal model (Wang et al. 2017).

Beyond therapeutic studies, porcine salivary glands have been engineered for systemic or environmental applications. For instance, transgenic pigs expressing phytase in the parotid glands secrete the enzyme into saliva, improving dietary phosphorus utilization and reducing manure-related environmental pollution (Golovan et al. 2001). Such studies provide insights into protein targeting, secretion pathways, and promoter specificity, illustrating the versatility of genetically modified pigs as models for both human disease research and innovative biotechnological applications.

In addition to viral-mediated gene transfer and genome editing, other regenerative strategies have been explored in salivary gland research, particularly in relation to stem cells (Lombaert et al. 2017; Jeon et al. 2024). Sui et al. (2020) demonstrated that human submandibular gland stem cells form functional salivary organoids in vitro and in vivo, exhibiting enhanced differentiation when exposed to FGF10 and embryonic mesenchyme (Sui et al. 2020). Similarly, Maria et al. (2011) demonstrated that culturing human submandibular gland (HSG) cells on Matrigel promoted their morphological and functional differentiation into polarized acinar-like cells, characterized by increased transepithelial electrical resistance, tight junction formation, and secretion of acinar markers such as α-amylase and aquaporin-5. These findings highlight the critical role of the extracellular matrix in promoting acinar phenotype and functional maturation, reinforcing the relevance of microenvironmental cues in salivary gland tissue engineering (Maria et al. 2011).

Bioprinting approaches have also emerged as promising strategies in this context. Bioprinting approaches have also emerged as promising strategies in this field. For instance, Adine et al. (2018) employed 3D magnetic bioprinting (M3DB) to generate epithelial organoids from human dental pulp stem cells (hDPSCs), which secreted α-amylase in response to FGF10 and promoted epithelial and neuronal growth upon transplantation (Adine et al. 2018). These studies contribute to the broader efforts to develop bioprinted salivary gland models with regenerative potential, as reviewed by Klangprapan et al. (2024).

## Conclusion

The literature on porcine salivary glands is yet scarce. However, the analysis of the few studies available on porcine salivary glands indicate they may serve as valuable models for studying human diseases due to their structural and functional similarities to human tissues. Their use in biomedical research has provided crucial insights into disease mechanisms, therapeutic interventions, and tissue engineering strategies. Despite their potential, further studies are needed to refine these models and address species-specific differences that may impact translational applications. Collaborative efforts between veterinary and medical researchers will be key to maximizing the utility of porcine salivary glands in advancing human health research. By leveraging these models, the scientific community can accelerate the development of innovative treatments and improve our understanding of complex human diseases.

## Data Availability

No datasets were generated or analysed during the current study.
